# Exploring the Mitochondrial Degradome by the TAILS Proteomics Approach in a Cellular Model of Parkinson’s Disease

**DOI:** 10.3389/fnagi.2019.00195

**Published:** 2019-07-31

**Authors:** Marta Lualdi, Maurizio Ronci, Mara Zilocchi, Federica Corno, Emily S. Turilli, Mauro Sponchiado, Antonio Aceto, Tiziana Alberio, Mauro Fasano

**Affiliations:** ^1^Department of Science and High Technology, University of Insubria, Busto Arsizio, Italy; ^2^Department of Pharmacy, University “G. D’Annunzio” of Chieti-Pescara, Chieti, Italy; ^3^Department of Biochemistry, Research and Innovation Centre, University of Regina, Regina, SK, Canada; ^4^Department of Medical, Oral and Biotechnological Sciences, University “G. D’Annunzio” of Chieti-Pescara, Chieti, Italy

**Keywords:** proteomics, degradomics, TAILS N-terminomics, mitochondria, Parkinson’s disease

## Abstract

Parkinson’s disease (PD) is the second most frequent neurodegenerative disease worldwide and the availability of early biomarkers and novel biotargets represents an urgent medical need. The main pathogenetic hallmark of PD is the specific loss of nigral dopaminergic neurons, in which mitochondrial dysfunction plays a crucial role. Mitochondrial proteases are central to the maintenance of healthy mitochondria and they have recently emerged as drug targets. However, an exhaustive characterization of these enzymes and their targets is still lacking, due to difficulties in analyzing proteolytic fragments by bottom-up proteomics approaches. Here, we propose the “mitochondrial dimethylation-TAILS” strategy, which combines the isolation of mitochondria with the enrichment of N-terminal peptides to analyze the mitochondrial N-terminome. We applied this method in a cellular model of altered dopamine homeostasis in neuroblastoma SH-SY5Y cells, which recapitulates early steps of PD pathogenesis. The main aim was to identify candidate mitochondrial proteases aberrantly activated by dopamine dysregulation and their cleaved targets. The proposed degradomics workflow was able to improve the identification of mitochondrial proteins if compared to classical shotgun analysis. In detail, 40% coverage of the mitochondrial proteome was obtained, the sequences of the transit peptides of two mitochondrial proteins were unveiled, and a consensus cleavage sequence for proteases involved in the processing of mitochondrial proteins was depicted. Mass spectrometry proteomics data have been submitted to ProteomeXchange with the identifier PXD013900. Moreover, sixty-one N-terminal peptides whose levels were affected by dopamine treatment were identified. By an in-depth analysis of the proteolytic peptides included in this list, eleven mitochondrial proteins showed altered proteolytic processing. One of these proteins (i.e., the 39S ribosomal protein L49 – MRPL49) was cleaved by the neprilysin protease, already exploited in clinics as a biotarget. We eventually demonstrated a mitochondrial subcellular localization of neprilysin in human cells for the first time. Collectively, these results shed new light on mitochondrial dysfunction linked to dopamine imbalance in PD and opened up the possibility to explore the mitochondrial targets of neprilysin as candidate biomarkers.

## Introduction

Among neurodegenerative diseases, Parkinson’s disease (PD) is the second most frequent worldwide and a huge health challenge for society. PD is primarily characterized by the specific loss of nigral dopaminergic neurons, accompanied by the onset of cardinal motor symptoms (Obeso et al., 2017). Current medical treatments can only aim at reducing symptoms, while early diagnostic tools are not available, which would actually impact on PD patients’ cure. Thus, a deep investigation of the earliest pathogenetic mechanisms in PD will lead to the discovery of reliable and measurable drug targets and biomarkers, which is an urgent medical need.

Since neurons are terminally differentiated cells that completely rely on oxidative phosphorylation for their energy supply, mitochondrial dysfunction plays a major role in neuronal cells death (Strauss et al., 2005; Shanbhag et al., 2012). Dopaminergic neurons are more sensitive to mitochondrial impairment than other neuron types since dopamine (DA) itself can undergo spontaneous oxidation when not correctly stored in acidic vesicles, thus fostering mitochondrial damage and oxidative stress (Subramaniam and Chesselet, 2013). For this reason, DA homeostasis alteration is recognized as an early pathogenetic event in PD (Hastings, 2009; Zilocchi et al., 2018). In this context, the human neuroblastoma SH-SY5Y cell line is a widely used cellular model to study PD pathogenesis, since a marked cytoplasmic accumulation of DA can be reached upon the addition of exogenous DA to the culture medium (Alberio et al., 2012). Using this cellular model, we have already demonstrated that alterations in DA homeostasis are related to an aberrant function of mitochondrial proteases (Alberio et al., 2014a,b; Di Pierro et al., 2016).

A highly conserved system of mitochondrial proteases exists, which ensures the maintenance of proper mitochondrial functions, both in healthy and stress conditions (Koppen and Langer, 2007). Indeed, these enzymes are central players in the mitochondrial unfolded protein response (mtUPR) that, together with fusion, fission, and mitophagy, is one of the main processes that restore mitochondrial proteostasis upon stress conditions. Beyond the degradation of misfolded or damaged proteins, mitochondrial proteases modulate many biochemical activities that are essential for mitochondrial function, such as mitochondrial dynamics, the apoptotic process, the maturation and import of proteins within mitochondria, and eventually their localization and function (Quirós et al., 2015). Consistent with their essential roles, mutations and/or functional alterations of these enzymes are linked to several human diseases, among which are cancer, multisystem diseases, and neurodegenerative disorders (López-Otín and Bond, 2008). Of note, some mitochondrial proteases seem to be implicated in PD pathogenesis. Indeed, mutations in the *HTRA2* (*PARK13*) gene, encoding a mitochondrial serine peptidase, promote the accumulation of alpha-synuclein deposits in mitochondria (Strauss et al., 2005). Moreover, mutations in *HTRA2* cause hereditary essential tremor and in homozygotes may result in the development of PD (Unal Gulsuner et al., 2014).

Very recently, mitochondrial proteases are emerging as potential pharmacological targets (Gibellini et al., 2016). Specific inhibitors have been developed and used to target those proteases whose overexpression or gain of function is associated with a specific disease. As an example, in a recent study the inhibition of the Lon protease homologue (LONP) by a synthetic triterpenoid resulted in lymphoma cells death (Bernstein et al., 2012), opening up to a potential use of this inhibitor in the treatment of tumors in which LONP is upregulated.

Despite their importance in both physiological and pathological processes, the cleavage activity of mitochondrial proteases and their protein targets are largely uncharacterized so far. Protease-generated fragments are indeed small and very low abundant peptides, semi-tryptic if trypsin is used for protein digestion, usually neglected by classical shotgun proteomics approaches. Indeed, after tryptic digestion these fragments often do not generate enough peptides for confident identification (Yates et al., 2009). To overcome this issue, specific proteomics procedures have been developed for degradomics analysis (Monti et al., 2019). These techniques are able to enrich and characterize the so-called “N-terminome,” which comprises both genetically determined N-termini of mature unprocessed proteins and “neo” N-termini, generated by proteolytic cleavages. Three main approaches have been developed for N-terminomics studies: combined fractional diagonal chromatography (COFRADIC), subtiligase-based enrichment of N-termini, and terminal amine isotopic labeling of substrates (TAILS). The first employs chemical modifications to alter the HPLC elution profiles of the tryptic peptides (Gevaert et al., 2003), the second is based on the positive selection (capture) of biotinylated N-terminal peptides (Mahrus et al., 2008), while the third involves the negative selection of previously labeled N-terminal peptides (Kleifeld et al., 2010b). TAILS is a high-throughput quantitative proteomic platform for protease substrate discovery and “N-terminome” analysis (Kleifeld et al., 2010a, 2011). The key steps in this procedure are the isotopic labeling (e.g., by reductive dimethylation), the enrichment (by negative selection) and the recovery of the N-terminal peptides, prior to perform the mass spectrometry analysis. The main application of the dimethylation-TAILS protocol is the identification of the complete set of protein targets for a specific protease of interest.

In the present work, we developed a new degradomics strategy, based on the dimethylation-TAILS approach, so to obtain a comprehensive characterization of the mitochondrial N-terminome in a model of altered DA homeostasis in SH-SY5Y cells. This cellular model mimics the early steps in PD pathogenesis and represents a great chance for the discovery of new candidate pathogenetic mechanisms and/or drug targets.

## Materials and Methods

### Cell Culture and Treatments

The human neuroblastoma SH-SY5Y cells (ECACC General Collection, cat# 94030304) were maintained at 37°C in a 5% CO_2_ humidified atmosphere and cultured in Dulbecco’s modified Eagle’s medium (DMEM), supplemented with 10% (v/v) heat-inactivated fetal bovine serum (FBS), 2 mM L-glutamine and antibiotics (100 U/ml penicillin and 100 μg/ml streptomycin). All cell culture media and reagents were from Euroclone (Pero, Milano, Italy). Cells were subcultured twice weekly and assessed to be mycoplasma-free. All treatments were performed between passages 6 and 9. Cells were seeded in five 225 cm^2^ flasks for control (CTRL) and DA-treated (DA) conditions, respectively. CTRL: 700 U/ml catalase (Sigma-Aldrich) in complete culture medium, 24 h. DA: 250 μM dopamine (Sigma-Aldrich) in CTRL medium, 24 h. Catalase was added to avoid the effects of extracellular oxidation of dopamine. Seven biological replicates were obtained.

### Isolation of Mitochondria

A commercial kit based on surfactants was used to isolate mitochondria from SH-SY5Y cells after treatment (MITOISO2 cell mitochondria isolation kit, Sigma-Aldrich). Isolation was performed following the manufacturer’s instructions. Briefly, CTRL and DA cells were detached by scraping and collected, washed twice in sterile ice-cold PBS and centrifuged. Cell count was performed so to start the isolation from 80 to 90 × 10^6^ total cells per sample. An aliquot of intact cells was frozen and stored for Western blot (WB) analysis. Cells were lysed following the instructions and incubated 5 min in ice, gently shaking. Vital count was performed to verify cell lysis. Two volumes of extraction buffer were added and cell lysates were centrifuged (600 × *g*, 10 min, 4°C) to obtain the pellet of nuclei and intact cells, which was washed in extraction buffer, centrifuged, fast-frozen in liquid nitrogen and stored for WB analysis. The supernatant was collected in a new tube and centrifuged (11000 × *g*, 10 min, 4°C) to obtain the pellet of mitochondria that was divided in two aliquots (one for TAILS, one for WB analysis), washed in extraction buffer, centrifuged and fast-frozen in liquid nitrogen. The supernatant, corresponding to the cytoplasmic fraction, was also stored.

### SDS-PAGE and Western Blotting

TAILS quality control SDS-PAGE was performed using the TGX Stain-Free FastCast Acrylamide Kit, 12% (Bio-Rad) and protein bands were visualized, captured and quantified with an UVP acquisition system (GelDoc-It^TM^ 310).

For WB analysis, cellular and/or organelle pellets derived from the isolation procedure were lysed in RIPA buffer (1% Triton X-100, 0.1% SDS, 1% sodium deoxycholate in 1.5 M Tris–HCl pH 7.4) added with protease inhibitors cocktail (Sigma-Aldrich) and incubated 30 min at 4°C in constant shaking. Lysates were mechanically sheared by sonication and cleared by centrifugation (12000 × *g*, 20 min, 4°C). Protein concentration in the supernatants was determined by the bicinchoninic acid assay (Thermo Fisher Scientific). Protein extracts (20–40 μg) were denatured in Laemmli buffer for 5 min at 95°C and separated on 10–16% SDS-PAGE gels. Proteins were transferred to PVDF membranes at 1 mA/cm^2^ for 1.5 h (TE77pwr, Hoefer). Membranes were blocked in 5% non-fat dried milk in TBS-T (0.1 M Tris–HCl pH 7.4, 1.5 M NaCl, 0.5% Tween-20) and incubated with the primary antibodies diluted in the same blocking solution, overnight at 4°C in constant shaking. Primary antibodies: rabbit polyclonal anti-VDAC1 (Abcam, ab15895), 1:1000 dilution; mouse monoclonal anti-citrate synthase (Sigma-Aldrich, AMAb91006), 1:1000 dilution; rabbit polyclonal anti-histone H3 (Sigma-Aldrich, H0164), 1:2500 dilution; mouse monoclonal anti-beta tubulin (Thermo Fisher Scientific, MA5-16308), 1:10000 dilution; rabbit polyclonal anti-neprilysin/CD18 (NovusBio, NBP2-15771), 1:1000 dilution; rabbit polyclonal anti-MRPL49 (NovusBio, NBP1-68932), 1:1000 dilution; mouse monoclonal anti-beta actin (Abcam, ab8226), 1:8000 dilution. After incubation with primary antibodies membranes were washed three times in TBS-T and incubated with peroxidase-conjugated secondary antibodies diluted in blocking solution. Secondary antibodies: Pierce^®^ goat anti-mouse IgG antibody (Thermo Fisher Scientific, 31432), 1:1000–1:8000 dilution; goat anti-rabbit IgG antibody (Millipore, AP132P), 1:1000–1:10000 dilution. Chemiluminescence detection was performed following manufacturer’s instructions (Millipore, WBKLS0500). Images (16 bit TIF grayscale) were acquired with the G:BOXChemi XT4 system (Syngene, Cambridge, United Kingdom) and analyzed using the ImageJ software.^1^ Signal intensities were corrected for protein loading by normalization to β-actin and/or β-tubulin intensities, or total protein amount per lane. Statistical significance was verified by either two-tailed *t*-test or two-way ANOVA.

### Mitochondrial Dimethylation-TAILS

**FIGURE 1:**
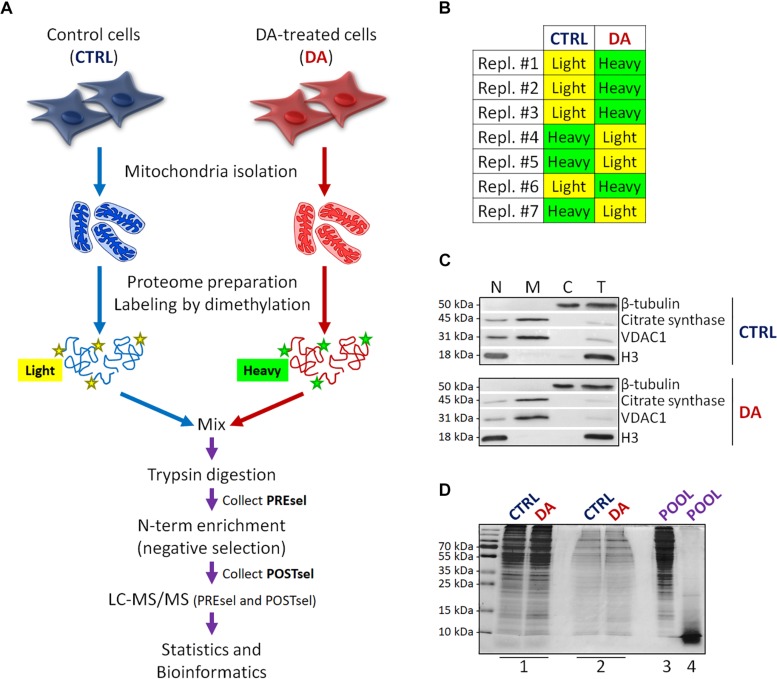
Mitochondrial dimethylation-TAILS. **(A)** Outline of the experimental workflow designed to investigate mitochondrial proteases and their substrates in a cellular model of altered DA homeostasis. After dopamine treatment, mitochondria were isolated from both control (CTRL) and DA-treated (DA) SH-SY5Y cells, isotopic labeling of primary amines was performed by either light or heavy dimethylation, samples were mixed, proteins were digested by trypsin and N-terminal peptides were enriched by negative selection, prior to perform the LC-MS/MS analysis. Both pre- (PREsel) and post- (POSTsel) negative selection samples have been collected and analyzed. **(B)** Table representing the labeling strategy for the seven biological replicates. **(C)** Representative WB analysis of fractions collected after mitochondria isolation. N, nuclei; M, mitochondria; C, cytoplasm; T, total extract. **(D)** TAILS quality control SDS-PAGE representing one in seven biological replicates. 1: mitochondrial proteins after lysis and first precipitation; 2: after isotopic labeling; 3: after pooling and second precipitation; 4: after trypsin digestion. See “Materials and Methods” section for further details.

The dimethylation-TAILS bench protocol was applied as described by Kleifeld et al. (2010b, 2011), with some modifications. All reagents were from Sigma-Aldrich, when not otherwise specified. Mitochondrial fractions were lysed in RIPA buffer added with protease inhibitors and cleared by high-speed centrifugation. Incubation with benzonase enzyme was performed to shear genomic DNA (15 min, room temperature). Proteins were extracted by acetone-methanol precipitation, the protein pellet was air dried and resuspended in 200 mM HEPES pH 8.0 (added with protease inhibitors) to achieve a final protein concentration of 2 mg/ml. Protein concentration was determined spectrophotometrically by both relative (BCA and/or Bradford assays) and absolute ([]⁢(mgml)=Abs⁢ 280⁢nm1,3⁢mlmg×cm× 1⁢cm) quantification methods. A total amount of 1 mg proteins per sample (CTRL and DA) was collected to perform the isotopic labeling. A small aliquot was stored for the TAILS quality control SDS-PAGE (Figure 1D, lane #1).

Proteins were then denatured with 4 M guanidinium chloride (GuHCl), reduced with 5 mM dithiothreitol (DTT) (1 h incubation at 37°C) and alkylated with 15 mM iodoacetamide (IAA) (30 min incubation at room temperature, in the dark). The excess IAA was quenched by the further addition of 15 mM DTT (20 min incubation at room temperature). Then, α-amines of both mature N-term and neo N-term, and ε-amines of lysine residues were blocked by reductive dimethylation. To this end, isotopic labeling was performed, using either ^12^CH_2_(light)-formaldehyde or ^13^CD_2_(heavy)-formaldehyde for CTRL and DA samples, respectively. Forty millimolar formaldehyde was added, followed by 20 mM sodium cyanoborohydride (NaBH_3_CN) and samples were incubated overnight at 37°C. An additional 2 h labeling was performed by adding 20 mM formaldehyde and 10 mM NaBH_3_CN. To quench the reaction, 100 mM Tris–HCl pH 6.8 was added (1 h incubation at 37°C). Small aliquots were stored at this point for the quality control SDS-PAGE (Figure 1D, lane #2).

CTRL and DA samples were then quantified and mixed in a single pool in 1:1 ratio, proteins were extracted by methanol-chloroform precipitation and the protein pellet was air dried and resuspended in 50 mM HEPES pH 8.0 to achieve a final protein concentration of 1 mg/ml. An aliquot was stored for the quality control SDS-PAGE (Figure 1D, lane #3).

Proteolysis was then performed with mass-spectrometry grade trypsin (Promega), overnight at 37°C in gentle shaking. A final trypsin/proteins ratio of 1:100 was used (500 μg of total proteins were digested with 5 μg trypsin). An aliquot was stored for the quality control SDS-PAGE (Figure 1D, lane #4). An additional 4 h incubation was performed by adding new trypsin (1:100).

Negative selection of blocked N-terminal peptides was then performed with the HPG-ALDII polymer developed by Kleifeld and colleagues,^2^ which covalently binds tryptic peptides containing free primary amines. A “Pre-negative selection” sample was stored (50 μg) to be analyzed by LC-MS/MS, so to determine the efficiency of the negative selection procedure in enriching the N-terminal peptides. Negative selection was performed by adding the polymer to the peptide mixture with a 5:1 ratio (w/w), followed by the addition of 20 mM NaBH_3_CN (overnight incubation at 37°C). To quench the reaction, 100 mM Tris–HCl pH 6.8 was added (1 h at 37°C). Ultrafiltration was performed following manufacturer’s instructions (Vivaspin 500, 10 kDa MWCO, Sartorius) so to retain the polymer and collect the unbound blocked N-terminal peptides in the ultrafiltrate. The retrieved peptides were then purified and concentrated [solid phase extraction (SPE) C18-SD cartridges, Empore] and eventually dried by evaporating the eluent (50% acetonitrile, 0.1% trifluoroacetic acid in water) in a SpeedVac.

### LC-MS/MS

Dried peptides were resuspended in 30 uL of 0.1% formic acid and analyzed as previously reported (Ronci et al., 2018). Four microliters per run were analyzed by nano LC-MS/MS using a Proxeon EASY-NlcII (Thermo Fisher Scientific) coupled to the maXis HD UHR-TOF mass spectrometer (Bruker Daltonics). Peptides were loaded on the EASY-Column C18 trapping column (2 cm L., 100 μm I.D, 5 μm ps, Thermo Fisher Scientific), and subsequently separated on an Acclaim PepMap100 C18 (75 μm I.D., 25 cm L, 5 μm ps, Thermo Fisher Scientific) nano scale chromatographic column. The flow rate was set to 300 nL/min and the gradient was from 3 to 35% of B in 80 min followed by 35–45% in 10 min and from 45 to 90% in 11 min. Mobile phase A was 0.1% formic acid in H_2_O and mobile phase B was 0.1% formic acid in acetonitrile. The mass spectrometer, typically providing 60000 FMHW resolution throughout the mass range, was equipped with a nanoESI spray source. The mass spectrometer was operated in positive ion polarity and Auto MS/MS mode (data dependent acquisition – DDA), using N_2_ as collision gas for CID fragmentation. Precursors in the range 350–2200 m/z (excluding 1220.0–1224.5 m/z) with a preferred charge state +2 to +5 (excluding singly charged ions) and absolute intensity above 4706 counts were selected for fragmentation in a maximum cycle time of 3 s. After acquiring one MS/MS spectrum, the precursors were actively excluded from selection for 30 s. Isolation width and collision energy for MS/MS fragmentation were set according to the mass and charge state of the precursor ions (from 3 to 9 Da and from 21 to 55 eV). In-source reference lock mass (1221.9906 m/z) was acquired online throughout the runs.

### MS Data Analysis and Bioinformatics

Raw mass spectrometry data were analyzed using the PEAKS^®^ Studio 7.5 software (Zhang et al., 2012). Spectra were matched against the neXtProt database (NP_Human_6_2017) and false discovery rate (FDR) was set to 1% at the peptide-spectrum matches (PSMs) level, while FDRs at the protein and peptide levels were automatically calculated by the PEAKS software. The post-translational modification (PTM) profile was set as follows: cysteine carbamidomethylation (ΔMass: 57.02), light lysine and N-term dimethylation (ΔMass: 28.03), heavy lysine and N-term dimethylation (ΔMass: 34.06), methionine oxidation (ΔMass: 15.99), glutamine and asparagine deamidation (ΔMass: 0.98). Other search parameters were: semi-ArgC enzyme specificity, 10.0 ppm parent mass error tolerance, 0.05 Da fragment mass error tolerance, three maximum missed cleavages. MS1 quantification was performed, based on chromatogram extracted areas (XIC). By means of a routine specifically written in “R” (available on request) the obtained lists of identifications were filtered so to extract peptide sequences, PSMs and Areas (peptide sequence parsing, removal of duplicated peptides). Each biological replicate was analyzed separately to identify differentially represented peptides in DA vs. CTRL. The DA/CTRL ratio (either light/heavy or heavy/light, depending on the labeling strategy) was first calculated and log_2_ transformed. Both “quantitative” and “qualitative” differences were retained. A maximum of two missing values was accepted across the seven biological replicates. For quantitative analysis, the threshold for significance was set to 1.5 fold-change, with *p* < 0.05.

N-terminal peptides were annotated by the TopFIND ExploRer program,^3^ which retrieves general and position-specific information for a list of N-terminal peptides (Fortelny et al., 2015). Human mitochondrial proteins were defined as those present in the human MitoCarta2.0 database (Calvo et al., 2016). The BioVenn web application (Hulsen et al., 2008) was used to generate area-proportional Venn diagrams. The WebLogo tool (Crooks et al., 2004) was used to generate the consensus cleavage sequence of the internal proteolytic peptides. The “Search for specificity” tool of MEROPS (Rawlings et al., 2014) was used to retrieve the information of candidate proteases based on the sequence of the internal peptides.

### Neprilysin Knock-Down Experiments

SH-SY5Y cells were seeded in 12-well plates and transfected 12 h later with the Lipofectamine^®^ RNAiMAX Reagent (Thermo Fisher Scientific) using 100 nM siRNAs/well in serum-free medium, according to the manufacturer’s instructions. The following siRNAs purchased from Dharmacon were used: ON-TARGET plus non-targeting pool (cat# D-001810-10-05) and ON-TARGET plus human MME (4311) siRNA SMART pool (cat# L-005112-00-0005). 36 h after transfection, cells were treated with DA as already described. After 24 h treatment, cells were washed in ice-cold PBS and harvested for either WB or RT-qPCR analysis. Three independent knock-down experiments were performed.

### RNA Extraction and RT-qPCR

Total RNA was extracted from SH-SY5Y cells using the ReliaPrep^TM^ RNA cell miniprep system (Z6011, Promega, Milan, Italy) following the manufacturer’s instructions. Two micrograms of DNA-free total RNA were reverse transcribed into first-strand cDNA with random primers in a 20 μl final volume using the GoScript^TM^ reverse transcription system (Promega, A5000). The qPCR analysis was performed in technical triplicate using 40 ng of cDNA in 25 μl/well in 96-well plates, using the GoTaq^®^ qPCR master mix (Promega, A6001). The CFX-Connect^TM^ Real-Time PCR system (Bio-Rad) was used. Beta-actin (ACTB) was quantified as reference housekeeping gene. The amplification steps were set as follows: a first step at 95°C for 10 min, 40 cycles (95°C for 15 s, 60°C for 1 min) and a final dissociation step (95°C for 15 s, 60°C for 20 s, 95°C for 15 s). The relative expression levels of neprilysin and MRPL49 transcripts were calculated using the ΔΔCt method. Statistical significance was assessed by either paired two-tailed *t*-test or two-way ANOVA. Primers design was performed by using the “Pick primers” tool of NCBI Nucleotide and manually adjusted to avoid the amplification of undesired sequences and to have comparable melting temperatures and reaction efficiency. Primer pairs sequences:

ACTB Fw: 5′-CAGCCATGTACGTTGCTATCCAGG-3′;
ACTB Rev: 5′-AGGTCCAGACGCAGGATGGCATGG-3′;
MRPL49 Fw: 5′-GCTGGCGTAGCAGGTAAAGATGG-3′;
MRPL49 Rev: 5′-CACAGACTCCACAAACCTGGGGTA-3′;
NEP Fw: 5′-GGAGATCAGCCTCTCGGTCCTT-3′;
NEP Rev: 5′-GGCTCAGTGGTGGCATCCATGT-3′.

## Results

### Outline and Application of the Mitochondrial Dimethylation-TAILS Workflow

With the final goal to identify candidate mitochondrial proteases involved in early PD pathogenesis, we designed a new degradomics setup (Figure 1A) based on the dimethylation-TAILS N-terminomics approach and we applied it to a cellular model of altered dopamine homeostasis. First, we treated neuroblastoma SH-SY5Y cells with dopamine and we isolated the mitochondria-enriched subcellular fraction from both CTRL and DA-treated (DA) cells, by using a detergent-based separation method that we have already described as the most efficient in our cellular model (Alberio et al., 2017). In mitochondrial isolates, we verified the enrichment of a mitochondrial marker protein (citrate synthase), the absence of nuclear contamination (histone H3) and the effectiveness of DA treatment (decreased levels of VDAC1) (Alberio et al., 2014a,b), by WB analysis (Figure 1C). Then, we extracted the mitochondrial proteomes and we performed the isotopic labeling of primary amines by reductive dimethylation with either light or heavy formaldehyde. As described in Figure 1B, seven biological replicates have been produced, to minimize the effects of intrinsic biological variability. Moreover, labeling swap was used across the replicates, so to rule out any bias of labeling efficiency. After the blocking of primary amines, we quantified and pooled CTRL and DA samples, so to proceed with tryptic digestion and negative selection of the N-terminal peptides using the aldehyde-branched polymer developed for TAILS (Kleifeld et al., 2011). A small amount of the sample was stored before selection (PREsel), to be also analyzed by LC-MS/MS. After selection, free N-terminal peptides generated by trypsin digestion were covalently bound to the polymer, while blocked N-terminal peptides (POSTsel) were eventually recovered by ultrafiltration. Among these peptides were (i) mature N-terminal peptides of unprocessed proteins, (ii) mature N-termini after the removal of a transit/signal peptide, and (iii) neo N-terminal peptides, generated by proteolytic cleavages. We performed a TAILS quality control SDS-PAGE (Figure 1D), in order to assess quantity and integrity of proteins (and peptides) at each step of the protocol.

### Characterization of the Mitochondrial N-Terminome

Raw MS spectra of both PREsel and POSTsel samples (*n = 7*) were matched against the neXtProt database in order to identify peptides and their corresponding proteins (Supplementary Tables S1, S2). As for the POSTsel samples, only TAILS-labeled peptides were considered (N-term dimethylation was set as fixed modification) and the number of identifications obtained is reported in Supplementary Table S3.

By contrast, search parameters were adjusted for the analysis of PREsel samples, in order to include unlabelled peptides (free N-term generated by tryptic digestion) in the list of the identifications. In this way we were able to calculate the peptide recovery $(R=\frac{\#\,\mathrm{peptides}\,\mathrm{POSTsel}}{\#\,\mathrm{peptides}\,\mathrm{PREsel}})$ for each replicate. As an average across all replicates, 85% (0.85 ± 0.05 *SD*; *n = 7*) of peptides resulted to be retained by the polymer, which is in keeping with existing TAILS literature, where a 90% reduction in the total peptides content is estimated (Doucet et al., 2011; Schilling et al., 2011; Wilson et al., 2013).

**FIGURE 2:**
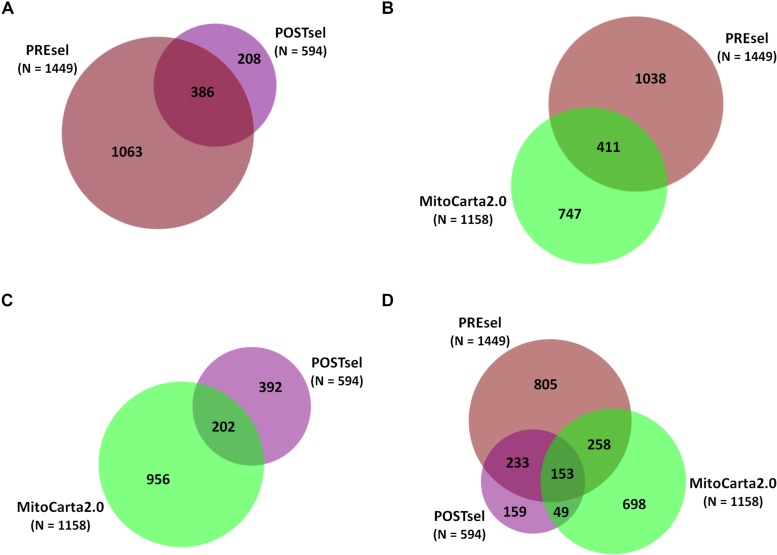
Protein identifications in PREsel and POSTsel. **(A)** Total proteins identified in PREsel (including tryptic peptides) and POSTsel (N-terminome alone) samples. **(B)** PREsel vs. human MitoCarta2.0 database (*n* = 1158). **(C)** POSTsel vs. MitoCarta 2.0. **(D)** Matching of the all identified proteins against MitoCarta2.0.

After the technical assessment of the negative selection procedure, we analyzed all proteins identified in both PREsel and POSTsel samples. Our aim was to assess whether mitochondrial enrichment coupled to the dimethylation-TAILS procedure was able to improve the success in identifying mitochondrial proteins. As shown in Figure 2A, we identified 1449 total proteins in PREsel samples, while 594 proteins were detected in POSTsel (386 proteins in common). Among the 1449 proteins in PREsel, 411 were identified as mitochondrial using human MitoCarta2.0 (Calvo et al., 2016) as the reference database (Figure 2B). On the other hand, 202 out of 594 proteins in POSTsel were mitochondrial (Figure 2C). We then calculated the fraction of mitochondrial proteins identified in both PREsel and POSTsel of each biological replicate. As a result, 29% (0.29 ± 0.02 *SD*) and 43% (0.43 ± 0.06 *SD*) of all proteins were identified as mitochondrial in PREsel and POSTsel samples, respectively. The significantly higher fraction of mitochondrial proteins obtained in POSTsel compared to PREsel (*p* = 0.0086, paired *t*-test) demonstrated that the application of the TAILS procedure was able to improve the identification of mitochondrial proteins. Strikingly, in POSTsel we also identified 49 mitochondrial proteins that were not present in PREsel (Figure 2D), further supporting the fact that the enrichment of the N-terminome represents a good strategy to study the mitochondrial proteome. Eventually, we obtained an overall 40% coverage of the mitochondrial proteome (460/1158 proteins).

After the analysis of all proteins identified in PREsel and POSTsel samples, we moved to an in-depth investigation of the N-terminome. In our POSTsel we identified 1083 total N-term peptides, belonging to 521 different proteins, among which 197 (38%) were mitochondrial. We analyzed the nature of these 1083 N-term peptides with TopFIND ExploRer (Fortelny et al., 2015) and we found that 570 peptides (53%) were TP-removal or internal N-term peptides (Supplementary Table S4). A list of 20 enriched proteases was identified by TopFINDer, in which four were mitochondrial, namely caspase-8 (CASP8, Q14790), mitochondrial-processing peptidase subunit beta (MPPB, O75439), serine protease HTRA2 (HTRA2, O43464), and methionine aminopeptidase 1D (MAP12, Q6UB28).

We then performed the same workflow with N-term peptides belonging to mitochondrial proteins only. The specific aims of this task were (i) analyzing the nature of the mitochondrial N-terminome, (ii) identifying candidate enriched proteases in our list, (iii) identifying a consensus cleavage sequence for proteases involved in the processing of mitochondrial proteins, and (iv) updating the human protein database with novel information from N-term peptides sequences. In general, the mitochondrial N-term peptides can be classified as (i) “natural N-term” of mature unprocessed proteins, (ii) “Met-removal N-term,” where the first methionine residue is removed, (iii) “mtTP-removal N-term,” where the mitochondrial transit peptide (mtTP) sequence is cleaved, and iv) “internal N-term,” representing the result of unpredicted proteolytic cleavages.

**FIGURE 3:**
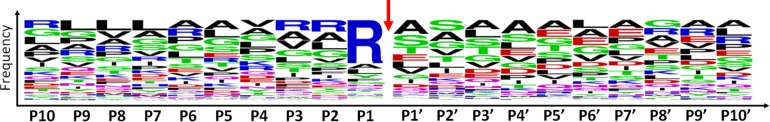
Consensus cleavage sequence for processed mitochondrial N-termini. Both mtTP-removal and internal N-term peptides (*n=438*) of mitochondrial proteins were analyzed to obtain the frequency plot. P1′ corresponds to the first aminoacid of the N-term peptide, while aminoacids P10-P1 represent the flanking region, up to the N-terminus of the protein. The red arrow represents the cleavage site.

Among the 479 peptides matching with mitochondrial proteins in POSTsel, 438 (91%) were identified as either mtTP-removal or internal N-term by TopFINDer (Supplementary Table S5). This high fraction was expected, since the vast majority of mitochondrial proteins undergoes post-translational processing to achieve the mature form. TopFINDer search identified eight proteases enriched in this list, including MPPB (O75439) and HTRA2 (O43464) as strictly mitochondrial ones. To depict the consensus cleavage sequence of proteases involved in the processing of mitochondrial proteins, we used WebLogo (Crooks et al., 2004). The aminoacidic sequence of each N-terminal peptide (P1′ to P10′) with its respective flanking region (P10 to P1) was analyzed. As shown in the aminoacid frequency plot (Figure 3), we identified Arg-Arg-Arg↓Ala-Ser as the consensus cleavage sequence.

Eventually, by an in-depth analysis of the identified mtTP-removal N-term peptides, we were able to define the precise sites of TP removal of two mitochondrial proteins (Threonine-tRNA ligase and 28S ribosomal protein S35), which are unidentified in the neXtProt database (Table 1 and Supplementary Figures S1, S2). The sequence information of these novel N-term peptides has already been submitted to neXtProt database curators for database update.

**TABLE 1:** Newly identified mtTP removal sites.

Protein	Identified N-term	**P′ position**	**mtTP**	**Chain**
Threonine-tRNA ligase (Q9BW92)	LHTAVVSTPPR	20	1–19	20–718
28S ribosomal protein S35 (P82673)	STAVYSATPVPTPSLPER	23	1–22	23–323

### Effects of Altered DA Homeostasis on Mitochondrial N-Terminome

After the overall characterization of the N-term peptides, we moved to the analysis of quantitative changes induced by DA treatment in the mitochondrial N-terminome. To this purpose, we calculated the ratio (log_2_ transformation) of the amounts of differentially labeled peptides (DA vs. CTRL) and we obtained the final list of N-terminal peptides, whose levels were significantly influenced by DA treatment. Both qualitative and quantitative changes were considered, since some peptides were present in both CTRL and DA with different amounts, while some others were present in DA only or viceversa. Overall, two missing values were accepted and the threshold for significance was set to 1.5 fold-change (±0.58 after log_2_ transformation). As a result, 61 N-term peptides were included in this list, grouped in two clusters: 24 peptides were over-represented in DA vs. CTRL (Table 2), while 37 showed the opposite trend (Table 3). Among peptides included in the first cluster, two were internal N-term, while the remaining 22 were either Met- or TP-removal N-term. Among the latter, three showed a one/two-amino acids shift when compared to the TP-removal sites reported in the UniProt database, likely generated by ragging aminopeptidase activity. On the other hand, among the 37 peptides under-represented in DA vs. CTRL, nine were internal, while 29 were either Met- or TP-removal N-term. Again, seven of them were generated by ragging peptidase activity.

**TABLE 2:** N-terminal peptides OVER-represented in DA vs. CTRL (*n=24*).

**N-term peptide sequence**	Protein^*^	Type of N-term	**DA/CTRL (log_2_) ± SEM**
SDMPPLTLEGIQDR	O14561	TP-removal	1.25 ± 0.44
LHLGVTPSVIR	O14773	TP-removal	DA only^∗∗^
STQAATQVVLNVPETR	O75439	“ragging” TP-removal	0.92 ± 0.26
SSEAPPLINEDVKR	P04843	“ragging” TP-removal	0.65 ± 0.05
DAPEEEDHVLVLR	P07237	TP-removal	0.98 ± 0.24
AKDVKFGADAR	P10809	TP-removal	0.72 ± 0.04
VNFTVDQIR	P13639	Met-removal	0.67 ± 0.07
DDEVDVDGTVEEDLGKSR	P14625	TP-removal	DA only^∗∗^
KEAESSPFVER	P14625	Internal	DA only^∗∗^
ASGANFEYIIAEKR	P30084	TP-removal	0.69 ± 0.08
SDVLELTDDNFESR	P30101	TP-removal	1.29 ± 0.45
DDLVTVKTPAFAESVTEGDVR	P36957	TP-removal	0.77 ± 0.14
VLLESEQFLTELTR	P37108	Met-removal	0.67 ± 0.09
DGVGGDPAVALPHR	P49257	TP-removal	1.09 ± 0.42
LRPGDCEVCISYLGR	P55145	TP-removal	DA only^∗∗^
SASVVSVISR	P61803	Met-removal	0.71 ± 0.08
ESETTTSLVLER	Q04837	TP-removal	0.75 ± 0.07
VMEKPSPLLVGR	Q13283	Met-removal	0.77 ± 0.05
LSQTQGPPDYPR	Q13405	Internal	0.80 ± 0.09
AAVAATAAAKGNGGGGGR	Q6Y1H2	Met-removal	0.67 ± 0.07
AATLILEPAGR	Q86TX2	Met-removal	0.67 ± 0.07
AADTQVSETLKR	Q92616	Met-removal	0.84 ± 0.19
RASPAGGPLEDVVIER	Q96AY3	“ragging” TP-removal	0.84 ± 0.16
SEVPGAAAEGSGGSGVGIGDR	Q9NPA0	TP-removal	0.79 ± 0.21

^*^ UniProt code. ^∗∗^ Qualitative change.

**TABLE 3:** N-terminal peptides UNDER-represented in DA vs. CTRL (*n=37*).

**N-term peptide sequence**	Protein^*^	Type of N-term	**DA/CTRL (log_2_) ± SEM**
VHQSVATDGPSSTQPALPKAR	O75251	“ragging” TP-removal	CTRL only^∗∗^
LAADVGKAGAER	P05141	Internal	−1.65 ± 0.60
GNLANVIR	P05141	Internal	CTRL only^∗∗^
AQTSPSPKAGAATGR	P06576	TP-removal	−0.70 ± 0.12
EICGPGIDIR	P08069	TP-removal	CTRL only^∗∗^
MAPEVLPKPR	P09669	Natural	−0.87 ± 0.26
APEVLPKPR	P09669	“ragging” TP-removal	−0.70 ± 0.12
TSTAPAASPNVR	P11498	“ragging” TP-removal	−0.72 ± 0.08
LAADVGKSGTER	P12236	Internal	−2.57 ± 0.67
NQSPVDQGATGASQGLLDR	P18827	Internal	−0.72 ± 0.08
SHGSQETDEEFDAR	P20674	TP-removal	−0.74 ± 0.15
VAHFTFQPDPEPR	P21953	TP-removal	CTRL only^∗∗^
VSKPTLKEVVIVSATR	P24752	TP-removal	−0.74 ± 0.11
QKTGTAEMSSILEER	P25705	TP-removal	−0.69 ± 0.10
AIAAKEKDIQEESTFSSR	P42704	TP-removal	−0.69 ± 0.08
ADCISEPVNR	P43304	TP-removal	−1.35 ± 0.27
SHTDIKVPDFSEYR	P47985	TP-removal	CTRL only^∗∗^
MKEGMSNNSTTSISQAR	P50150	Met-removal	−0.81 ± 0.15
APSVPAAEPEYPKGIR	P54819	Met-removal	−0.66 ± 0.05
SVPAAEPEYPKGIR	P54819	“ragging” TP-removal	CTRL only^∗∗^
GVQVETISPGDGR	P62942	Met-removal	−0.59 ± 0.16
STAVYSATPVPTPSLPER	P82673	TP-removal (new)	−0.71 ± 0.11
SETTTSLVLER	Q04837	“ragging” TP-removal	CTRL only^∗∗^
TVNSLNVSANEN	Q13740	Internal	CTRL only^∗∗^
AELPPVPGGPR	Q14249	TP-removal	CTRL only^∗∗^
STNVQKEGQGSQTLR	Q6PML9	Internal	−0.70 ± 0.06
GAPEVLVSAPR	Q7Z4H8	TP-removal	−0.73 ± 0.12
GGSCPHPSSAPR	Q8WY21	TP-removal	CTRL only^∗∗^
STLQEVVGIR	Q969P0	Internal	CTRL only^∗∗^
EEQPPETAAQR	Q96HE7	TP-removal	−0.68 ± 0.07
HGEEQPPETAAQR	Q96HE7	“ragging” TP-removal	CTRL only^∗∗^
SAPGEDEECGR	Q99523	Propeptide-removal	CTRL only^∗∗^
TTFNIQDGPDFQDR	Q99757	TP-removal	−0.72 ± 0.12
LHTAVVSTPPR	Q9BW92	TP-removal (new)	CTRL only^∗∗^
YSKSPSNKDAALLEAAR	Q9H078	Internal	CTRL only^∗∗^
LHTAANAAATATETTCQDVAATPVAR	Q9NP92	Internal	−0.72 ± 0.08
SCPGTVAKDLR	Q9NVV4	“ragging” TP-removal	CTRL only^∗∗^

^*^ UniProt code. ^∗∗^ Qualitative change.

**FIGURE 4:**
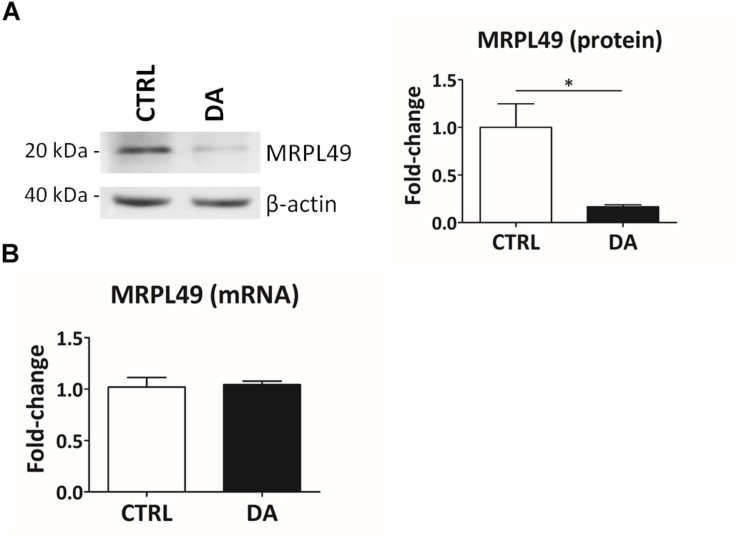
MRPL49 protein is proteolytically processed upon DA treatment. **(A)** Left: representative WB analysis showing the decrease in the levels of the MRPL49 protein upon DA treatment. Right: relative fold-change in the levels of MRPL49 protein in DA vs. CTRL. Four biological replicates (*n=4*). Error bars: SEM. Statistical analysis performed by paired two-tailed *t*-test. ^*^p = 0.036. **(B)** Relative fold-change in the levels of MRPL49 transcript by RT-qPCR. Three biological replicates (*n=3*). Error bars represent SEM. Statistical analysis performed by paired two-tailed *t*-test. *p* = 0.85.

Altogether the 61 peptides belonged to 55 proteins. In order to verify whether they took part to common biochemical pathways, we used the “analyze data” tool of the Reactome database (Fabregat et al., 2017). The over-represented pathways (*FDR* < 0.05) were “Respiratory electron transport, ATP synthesis by chemiosmotic coupling, and heat production by uncoupling proteins” (R-HSA-163200; *FDR* =  2.53 ×  10^−4^), “The citric acid (TCA) cycle and respiratory electron transport” (R-HSA-1428517; *FDR* =  2.53 ×  10^−4^), the “Respiratory electron transport” (R-HSA-611105; *FDR* = 0.002), the “Mitochondrial protein import” (R-HSA-1268020; *FDR* = 0.002), the “Vpr-mediated induction of apoptosis by mitochondrial outer membrane permeabilization” (R-HSA-180897; *FDR* = 0.013) and the “Detoxification of Reactive Oxygen Species” (R-HSA-3299685; *FDR* = 0.016). Moreover, we performed a TopFINDer search on both clusters of N-terminal peptides (*n = 61*), in order to identify putative enriched proteases whose activity was influenced by DA treatment (Supplementary Table S6). Since no candidate proteases were obtained, we decided to manually perform a MEROPS database search (Rawlings et al., 2014) for all the internal N-term peptides identified in the two clusters (11 peptides). Since our previous results suggested an hyperactivation of mitochondrial proteases upon DA treatment in SH-SY5Y cells (Alberio et al., 2014a,b; Di Pierro et al., 2016), we first focused our search on the two internal peptides significantly increased in DA-treated cells (see “internal” in Table 2). The first one (seq: KEAESSPFVER), scarcely interesting to us, belonged to endoplasmin, a molecular chaperone of the endoplasmic reticulum that functions in the processing and transport of secreted proteins. Conversely, the second one (seq: LSQTQGPPDYPR) matched with the mitochondrial 39S ribosomal protein L49 (MRPL49), a structural constituent of the mitochondrial ribosome (Supplementary Figure S3). Thus, we decided to verify if the overexpression of this internal peptide was indicative of an increased proteolytic processing of the corresponding protein. To this purpose, we assessed the total levels of the MRPL49 protein in DA vs. CTRL cells, by WB analysis (Supplementary Figure S4). As shown in Figure 4A, the levels of the MRPL49 protein resulted to be significantly decreased upon DA treatment. To rule out the hypothesis that the reduction of the full-length protein was due to decreased expression levels upon DA treatment, we also quantified the levels of MRPL49 transcript by RT-qPCR (Figure 4B). No significant change was observed at the mRNA level, thus demonstrating that MRPL49 protein reduction was due to proteolytic cleavage.

By means of MEROPS database search with the peptide sequence and the protein identifier, we found neprilysin (MEROPS identifier: M13.001) as the candidate human protease for the observed cleavage. This protease captured our attention because of its expression in brain tissue, its suggested mitochondrial localization in other species and its known involvement in neurodegenerative disorders (Bayes-Genis et al., 2016). No additional candidate proteases have been found by manual MEROPS search using the sequences of the other internal peptides influenced by DA.

### Validation of Neprilysin as a Protease Aberrantly Activated by Altered Dopamine Homeostasis

**FIGURE 5:**
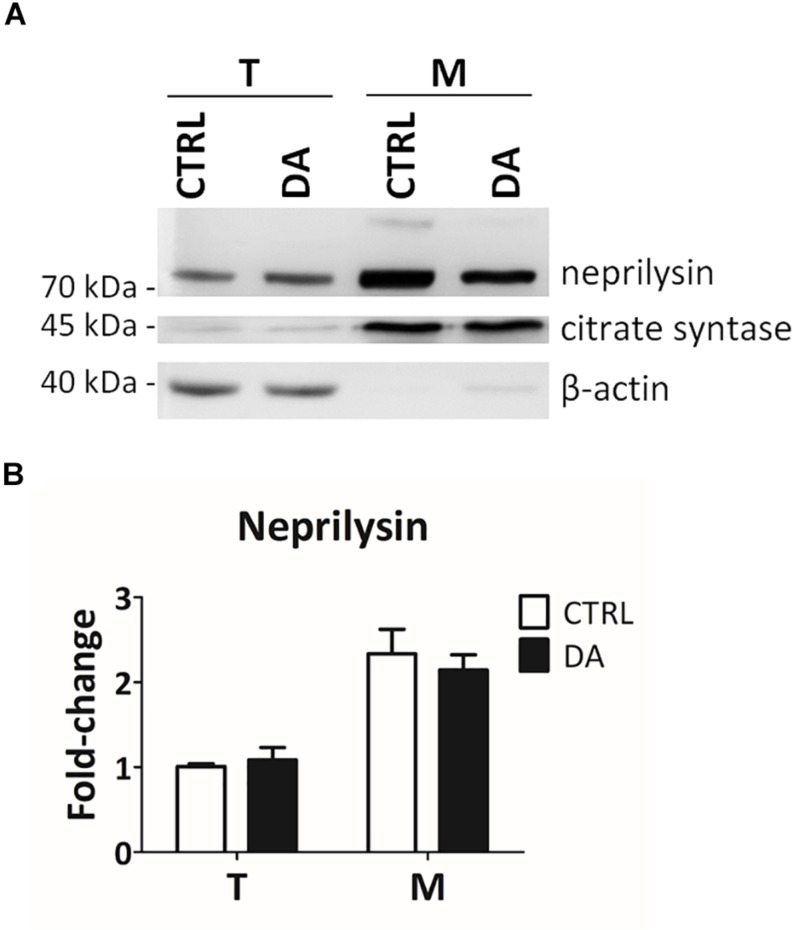
Neprilysin expression and localization in SH-SY5Y cells. **(A)** Representative WB analysis showing the presence of neprilysin in SH-SY5Y cells (T: total extracts) and its enrichment in mitochondrial isolates (M). **(B)** Relative fold-change in the levels of neprilysin in both total (T) and mitochondrial (M) extracts upon DA treatment, showing the significant enrichment of neprilysin in the mitochondrial fraction. Normalization was based on total protein amount per lane. Three biological replicates (*n=3*). Error bars: SEM. Statistical analysis performed by two-way ANOVA, to assess the effects of both “localization” (T vs. M) and “treatment” (CTRL vs. DA). The only significant source of variation was “localization” (*p* = 0.0002; *F* = 40.55).

In order to empirically verify *in silico* results about the possible proteolytic activity of neprilysin on MRPL49, we decided to (i) verify the expression of neprilysin in our cellular model, and (ii) assess its mitochondrial subcellular localization, by WB analysis (Supplementary Figure S5). As shown in Figure 5, neprilysin resulted to be expressed in SH-SY5Y cells and it was significantly enriched in the mitochondrial subcellular fraction (*p* = 0.0002; *F* = 40.55). We also verified whether any change in the levels of neprilysin was induced by DA treatment, but no significant differences were observed in DA vs. CTRL. This suggested that altered DA homeostasis could increase the activity but not the expression levels of neprilysin.

**FIGURE 6:**
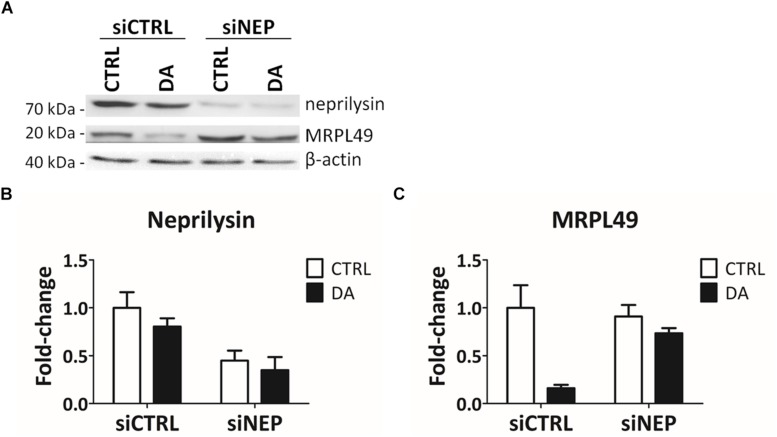
MRPL49 protein is a substrate of neprilysin. **(A)** Representative WB analysis showing the rescue of MRPL49 levels, after neprilysin knock-down (KD). siCTRL: pool of control siRNAs. siNEP: pool of neprilysin-silencing siRNAs. **(B)** Relative fold-change in the levels of neprilysin protein in both controls (siCTRL) and neprilysin-silenced (siNEP) cells upon DA treatment, showing the effectiveness of the KD. Three biological replicates (*n=3*). Error bars: SEM. Statistical analysis performed by two-way ANOVA, to assess the effects of both “KD” (siCTRL vs. siNEP) and “treatment” (CTRL vs. DA). The only significant source of variation was “KD” (*p* = 0.004; *F* = 15.98). **(C)** Relative fold-change in the levels of MRPL49 protein in siCTRL and siNEP cells upon DA treatment, showing the rescue of the levels of MRPL49 upon neprilysin KD. Three biological replicates (*n=3*). Error bars: SEM. Statistical analysis performed by two-way ANOVA. Both “treatment” (*p* = 0.0057; *F* = 14.02) and “interaction” (*p* = 0.040; *F* = 5.98) resulted to be significant sources of variation.

In the final attempt to directly demonstrate the activity of neprilysin as the protease responsible for the cleavage of MRPL49, we performed neprilysin knock-down (KD) in our cellular model. To this purpose, SH-SY5Y cells were transiently transfected with a pool of siRNAs (siNEP or siCTRL) and then treated with DA. The levels of both neprilysin and MRPL49 were assessed by WB (Supplementary Figure S6) and RT-qPCR analysis. As shown in Figure 6, we obtained an efficient KD of neprilysin at the protein level (siCTRL vs. siNEP, *p* = 0.004; *F* = 15.98), accompanied by a statistically significant rescue of the levels of MRPL49 protein upon DA treatment (both “treatment” (*p* = 0.0057; *F* = 14.02) and “interaction between KD and treatment” (*p* = 0.040; *F* = 5.98) resulted to be significant sources of variation). Neprilysin transcript levels (Supplementary Figure S7A) confirmed neprilysin KD, while no significant changes were observed for MRPL49 at the mRNA level upon both neprilysin KD and DA treatment (Supplementary Figure S7B). These results confirmed the actual role of neprilysin in the proteolytic cleavage of MRPL49 and also pointed out the role of neprilysin as a mitochondrial protease whose function is aberrantly activated by altered DA homeostasis.

## Discussion

PD affects four million people worldwide and this number is estimated to almost double within 2030. PD is complex and multifactorial in both etiology and clinical manifestations and patients can only count on symptomatic treatments (both pharmacological and surgical). In this framework, omics approaches, proteomics first, represent the most effective strategy to unveil hidden mechanisms underlying PD pathogenesis, thus leading to the discovery of novel candidate biotargets and biomarkers (Alberio and Fasano, 2011; Chen-Plotkin et al., 2018; Lualdi and Fasano, 2018).

Mitochondrial dysfunction is a well-known mechanism involved in dopaminergic neurons degeneration. Thus, many cellular and animal models have been established to investigate the role of mitochondria in PD pathogenesis (Hu and Wang, 2016). Nonetheless, an aspect that remains almost unexplored to date is the role played by mitochondrial proteases. These enzymes are of pivotal importance in the maintenance of healthy mitochondria, are involved in aging and disease states, and represent potential drug targets (Quirós et al., 2015; Gibellini et al., 2016). Due to difficulties in studying their activity by means of classical shotgun proteomics approaches, specific workflows have been proposed to enrich and analyze all the N-terminal peptides in a system (N-terminome), among which proteolytic fragments are more easily identifiable.

Here, we proposed a quantitative degradomics strategy that combines the isolation of the mitochondrial subcellular fraction with the dimethylation-TAILS approach to enrich N-terminal peptides. We applied this “mitochondrial dimethylation-TAILS” workflow to a cellular model of altered dopamine homeostasis in SH-SY5Y cells, that we have already extensively characterized in terms of both mitochondrial dynamics (Zilocchi et al., 2018) and proteome landscape (Alberio et al., 2014a, 2017; Di Pierro et al., 2016). The main advantages for us using this model were that (i) DA treatment conditions were consolidated, (ii) the best method to perform mitochondria isolation has already been established (Alberio et al., 2017), and (iii) this model has been demonstrated to better recapitulate what observed in PD patients’ substantia nigra (Zilocchi et al., 2018). Thus, the first part of our workflow was the same that we have used in our previous shotgun proteomics analysis, making it easier a comparison between results. As for the second part, the dimethylation-TAILS protocol is a relatively cost-effective setup and it was only slightly adjusted to be applied to our experimental model. Using this strategy, we were able here to obtain (i) a general characterization of the mitochondrial N-terminome in our cellular model, and (ii) a quantitative analysis of the effects of altered DA homeostasis on the N-terminome. This eventually led us to the identification of neprilysin as a candidate protease aberrantly activated upon DA treatment and involved in mitochondrial dysfunction linked to PD.

In proteomics literature, only two other papers reporting about mitochondrial N-terminomics have been published to date. Vaca Jacome et al. (2015) first performed free N-terminome analysis of human mitochondria-enriched samples using trimethoxyphenyl phosphonium (TMPP) labeling approache. They identified the natural N-terminus of 693 unique proteins and 302 new cleavage sites. Very recently, Marshall et al. (2018) developed the MS-TAILS (mitochondrial SILAC-TAILS) and applied it to HeLa cells to investigate early intrinsic apoptosis. They actually identified some cleavage events that are central in early intrinsic apoptosis. In the present work we reached a 40% coverage of the human mitochondrial proteome, that is comparable to that obtained by Marshall and colleagues. Of note, we identified for the first time an internal N-terminal peptide (LLDVDNR; P1′ position: 135) belonging to one of the thirteen proteins encoded by the mitochondrial genome, i.e., the cytochrome c oxidase subunit 2 (P00403). Among the 1083 N-terminal peptides that we identified in our POSTsel samples, 746 (69%) can be considered “new,” since there is no other experimental evidence for them in previous N-terminomics studies. If we focus on N-terminal peptides of mitochondrial proteins only (438 in total), the fraction of newly identified N-termini is 63% (278/438), which nicely contributes to increase the knowledge in the field of mitochondrial terminomics. Strikingly, we demonstrated that mitochondria isolation combined to N-terminome enrichment allows to improve the identification of mitochondrial proteins. More in general, this suggests that the enrichment of the N-terminome could be convenient to study the mitochondrial proteome, which is of great interest due to the involvement of these organelles in a plethora of physiological and pathological conditions.

By an in-depth analysis of the mitochondrial N-terminome, we established that 91% of the N-terminal peptides identified in our study were either mtTP-removal or internal N-terminal peptides, an evidence that testifies the high frequency of proteolytic processing of mitochondrial proteins if compared to other cellular compartments. Moreover, we identified a consensus cleavage sequence (Arg-Arg-Arg↓Ala-Ser) for proteases involved in processing of mitochondrial proteins, that confirmed the pattern firstly identified by Marshall and colleagues. Eventually, we were able to define the precise sites of mtTP-removal of two mitochondrial proteins (Threonine-tRNA ligase and 28S ribosomal protein S35), and to submit this information for the update of the neXtProt database.

As for the effects of altered DA homeostasis on the mitochondrial N-terminome, we obtained a list of 61 N-terminal peptides whose levels were significantly influenced by DA treatment. They belong to 55 proteins. Firstly, we verified that different levels of N-terms were not due to a general over- or under- representation of the whole protein. To do so we compared results with our previous shotgun proteomics analysis on the mitochondrial fraction of the same cellular model (Alberio et al., 2014a). For example, we already verified that endoplasmin was up-regulated after DA treatment. Since we observed higher levels of its N-terms, we can conclude that endoplasmin protein levels are higher after DA treatment. Among the internal N-terminal peptides (11 in total) differentially represented in DA vs. CTRL, we first focused our attention on those over-represented in DA-treated cells, due to the fact that we were looking for candidate proteases aberrantly activated by altered dopamine homeostasis as putative biotarget in PD. Starting from the sequence information of one of these internal peptides, belonging to the MRPL49 protein, we identified neprilysin as a candidate protease activated by altered DA homeostasis. Here, we demonstrated for the first time that the human neprilysin peptidase is enriched in mitochondrial subcellular fractions, an evidence that has already been proposed for orthologues in other species (Wilson et al., 2014; Grois et al., 2017) but that was not confirmed to date in human cells. We also demonstrated that the alteration of DA homeostasis, which is among the earliest events in PD pathogenesis, does not seem to be able to alter the levels of expression of neprilysin, whose activity seems instead to be influenced (increased) in our cellular model. We also experimentally validated the link between the target protein (MRPL49) and the protease (neprilysin) by performing neprilysin knock-down experiments, which actually demonstrated that MRPL49 is a mitochondrial protein target of neprilysin.

Based on this evidence, it would be tempting to propose neprilysin as a candidate biotarget in PD. However, speculating on this kind of a role is quite tricky. Neprilysin is indeed a zinc-dependent endopeptidase, ubiquitously distributed and endowed with a plethora of functions (Bayes-Genis et al., 2016). It exists as a transmembrane protease, but it can also be found as a soluble catalytically active protein in biological fluids, such as blood, urine and CSF. Among its known substrates are angiotensin, bradykinin, substance P, glucagon, insulin β-chain, amyloid-beta, enkephalins and oxytocin, thus spanning from cardio-renal to gastrointestinal, respiratory and nervous systems. Neprilysin is already recognized as a biotarget because of the benefits obtained using neprilysin inhibitors (NEPis; e.g., thiorphan) in combination with angiotensin 2 type 1 receptor blockade for the treatment of systolic heart failure (McMurray et al., 2014). However, the chronic use of NEPis -which are capable of crossing the blood brain barrier- raised some concerns, due to the main functions of the protease in the brain. Indeed, neprilysin co-localizes with opioid receptors in the structures related to pain control and it is also detectable in the caudate-putamen, globus pallidus and substantia nigra. The first synthetic NEPis were developed as potential analgesic agents (Roques et al., 1993), but the role of the peptidase in the cleavage of the amyloid-beta peptide is now recognized as the most central in the nervous system. Indeed, neprilysin activity is usually reduced in Alzheimer’s disease (AD), thus contributing to the deposition of amyloid plaques. In keeping with this, the injection of NEPis in mouse brains impaired cognitive functions (Grimm et al., 2013), while the introduction of the neprilysin gene with lentiviral vectors was able to rescue the phenotype (Iwata et al., 2001). However, the role of neprilysin in AD pathogenesis is quite controversial, since some recent works demonstrated that the reduction in plaque accumulation in mouse models was not accompanied by a reduction in amyloid-beta oligomers and by an improvement in cognitive deficits (Meilandt et al., 2009). Efforts have been recently made to develop neprilysin variants with increased affinity for amyloid-beta peptide, so to use neprilysin as pharmacological treatment.

As far as PD concerns, no evidence for a role of neprilysin in the pathogenesis of this disease can be found in current literature. In our opinion, too many roles in too many districts are in charge of this protein to propose it as a valuable biotarget. Moreover, the possible role as a biomarker is difficult to be proposed, since we only observed an aberrant activation of this protease in our cellular model of altered DA homeostasis, but this was not accompanied by a significant increase/decrease in its levels. Based on both current literature and the present work, the most plausible interpretation for our results is that neprilysin can be hyperactivated upon alterations of the normal DA homeostasis, thus contributing to mitochondrial dysfunction, which represents a crucial early event in the onset of both sporadic and familiar PD. More intriguing than the exploitation of neprilysin as biomarker/biorarget is, in our opinion, the possibility to explore the mitochondrial protein targets of neprilysin as crucial factors in mitochondrial dysfunction linked to DA imbalance. Further investigations aimed at identifying the complete set of mitochondrial substrates of neprilysin is thus mandatory, in order to obtain a list of candidates (in addition to MRPL49).

Overall, in the present work we proposed a degradomics workflow that proved to be useful to gain more insights in mitochondrial dynamics, which are of pivotal importance in the pathogenesis of PD and several other diseases. We also collected compelling evidence of an aberrant function of mitochondrial proteases upon DA homeostasis disruption and we identified for the first time neprilysin as a candidate mitochondrial protease hyperactivated in this condition.

## Data Availability

The datasets generated for this study can be found in the ProteomeXchange (Accession: PXD013900; PWD: cicciociccio).

## Author Contributions

ML, TA, and MF designed the experiments. ML, FC, ET, and MS carried out the “mitochondrial-dimethylation TAILS” experiments. MR and AA carried out the LC-MS/MS analysis. ML, MR, ET, and MS carried out the bioinformatics analysis. MF carried out the statistical analysis. ML and MZ carried out the experimental verification. ML wrote the manuscript. TA and MF revised the manuscript. All authors read and approved the final version of the manuscript.

## Conflict of Interest Statement

The authors declare that the research was conducted in the absence of any commercial or financial relationships that could be construed as a potential conflict of interest.

## Footnotes

1 http://rsb.info.nih.gov/ij/

2 https://www.flintbox.com/public/project/1948/

3 http://clipserve.clip.ubc.ca/topfind/topfinder
