# Supplementary material for: Exploring the Mitochondrial Degradome by the TAILS Proteomics Approach in a Cellular Model of Parkinson’s Disease
Source: Front Aging Neurosci. 2019 Jul 31;11:195. doi: 10.3389/fnagi.2019.00195 (PMC6685049; doi:10.3389/fnagi.2019.00195)
Supplement: Supplementary file 3 [file Table_3.docx]

**Supplementary Table 3. N-terminome identifications in POSTsel samples**

| Sample | #PSM | #peptides | #proteins |
| --- | --- | --- | --- |
| POSTsel Repl. #1 | 639 | 474 | 289 |
| POSTsel Repl. #2 | 1118 | 329 | 228 |
| POSTsel Repl. #3 | 1206 | 453 | 287 |
| POSTsel Repl. #4 | 528 | 161 | 125 |
| POSTsel Repl. #5 | 1901 | 733 | 463 |
| POSTsel Repl. #6 | 2489 | 918 | 620 |
| POSTsel Repl. #7 | 1600 | 482 | 338 |
