# Supplementary figures and images for: Exploring the Mitochondrial Degradome by the TAILS Proteomics Approach in a Cellular Model of Parkinson’s Disease
Source: Front Aging Neurosci. 2019 Jul 31;11:195. doi: 10.3389/fnagi.2019.00195 (PMC6685049; doi:10.3389/fnagi.2019.00195)

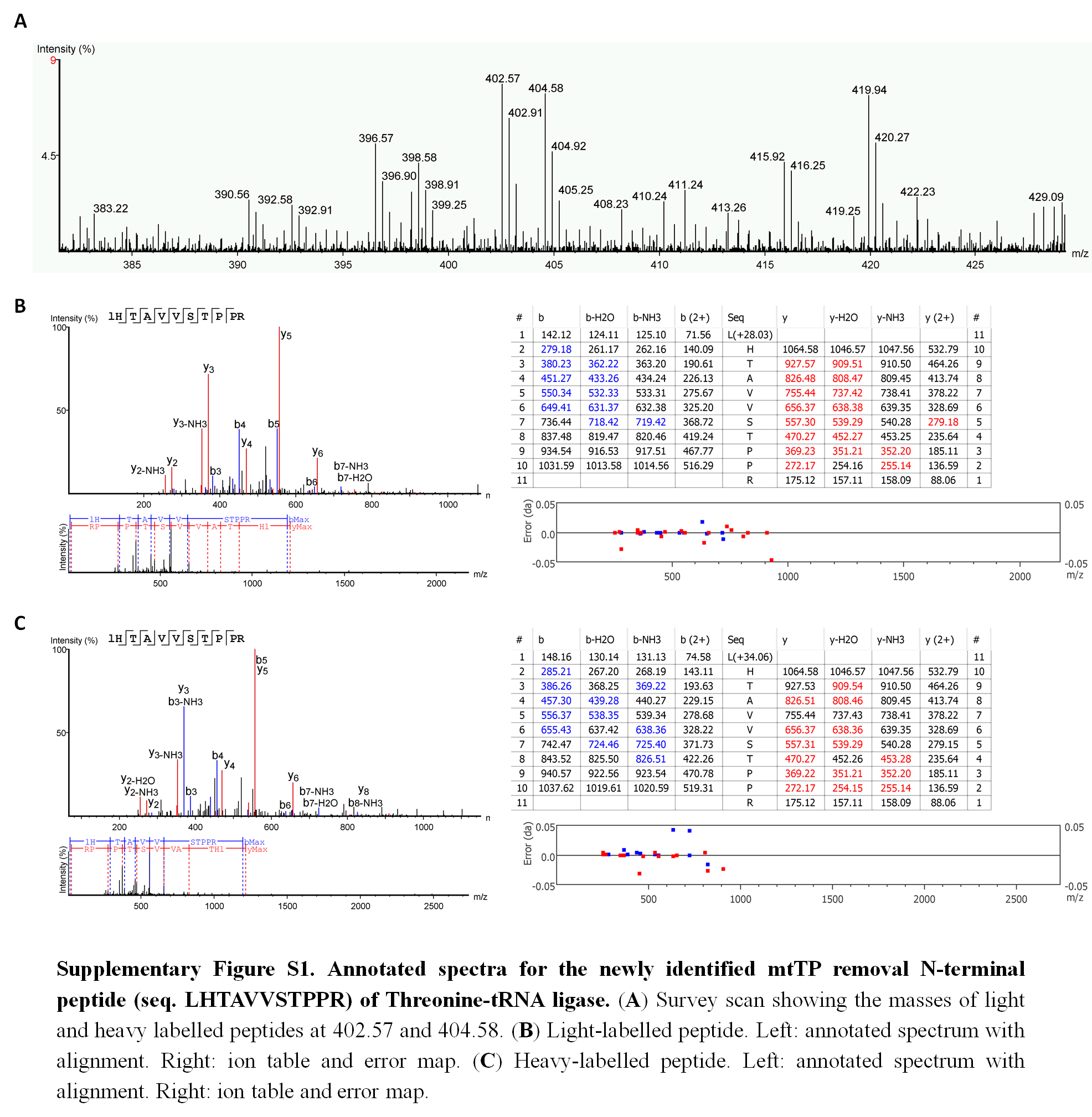

Supplement: Supplementary file 7 [file Image_1.tif]

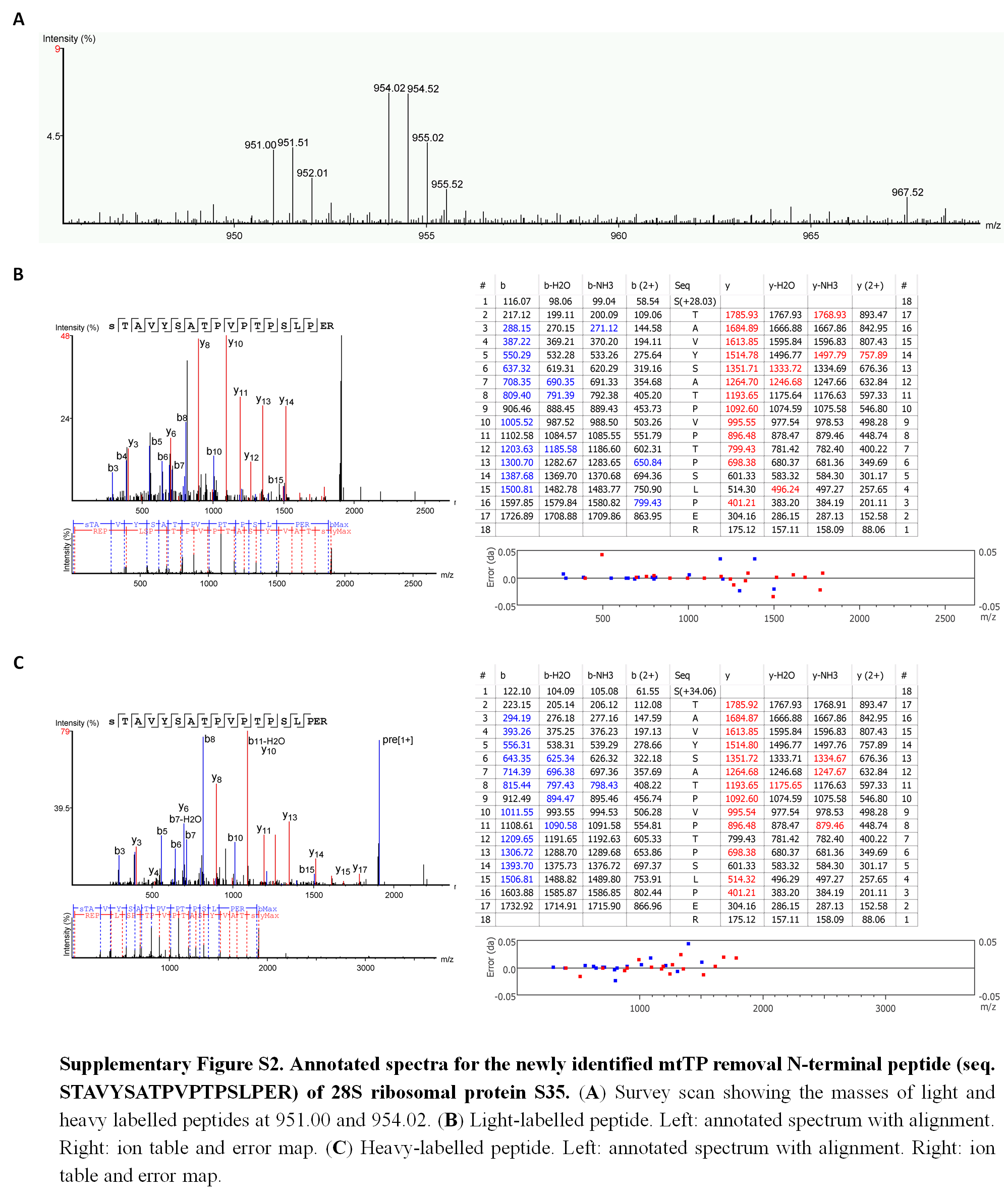

Supplement: Supplementary file 8 [file Image_2.tif]

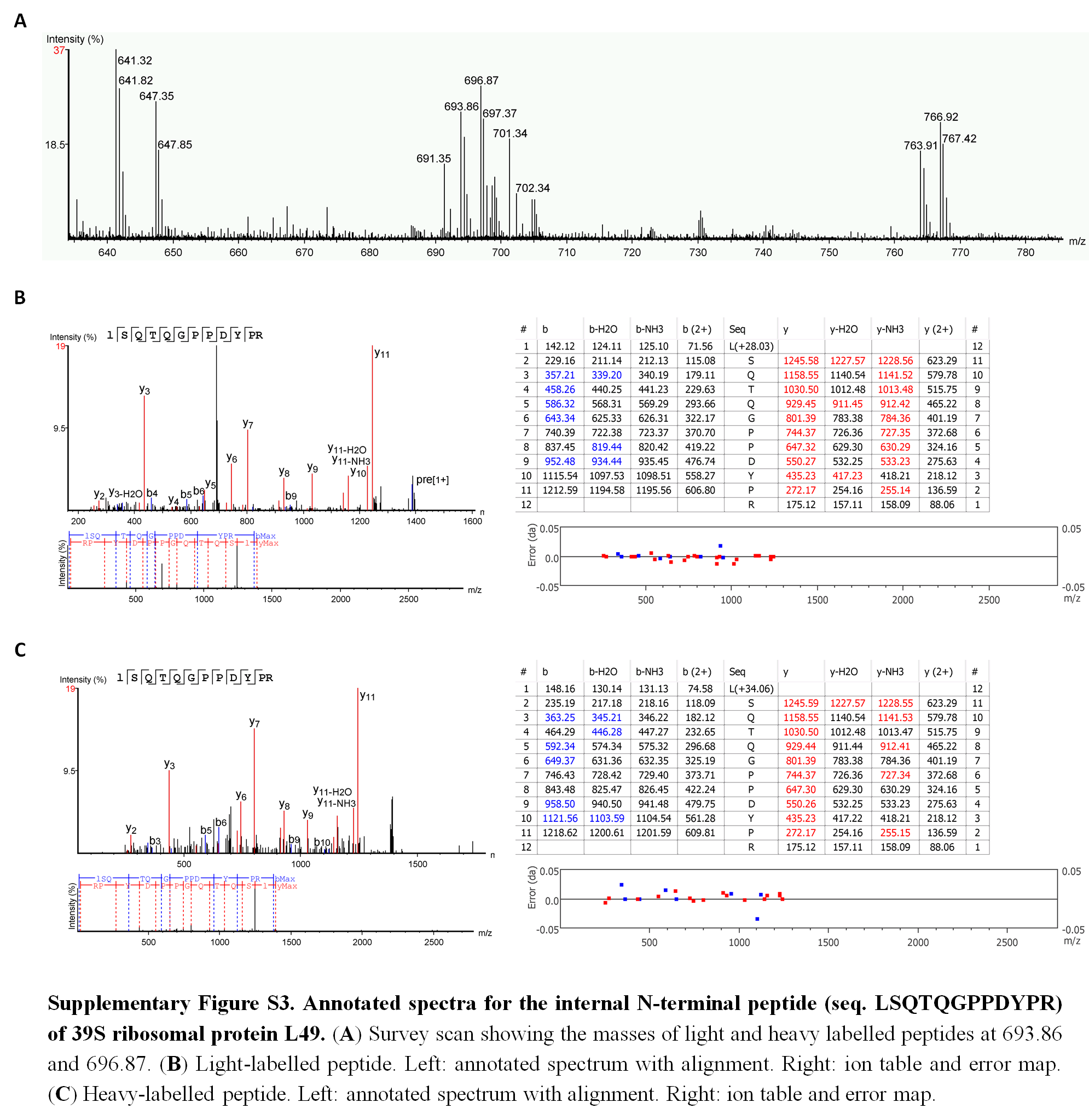

Supplement: Supplementary file 9 [file Image_3.tif]

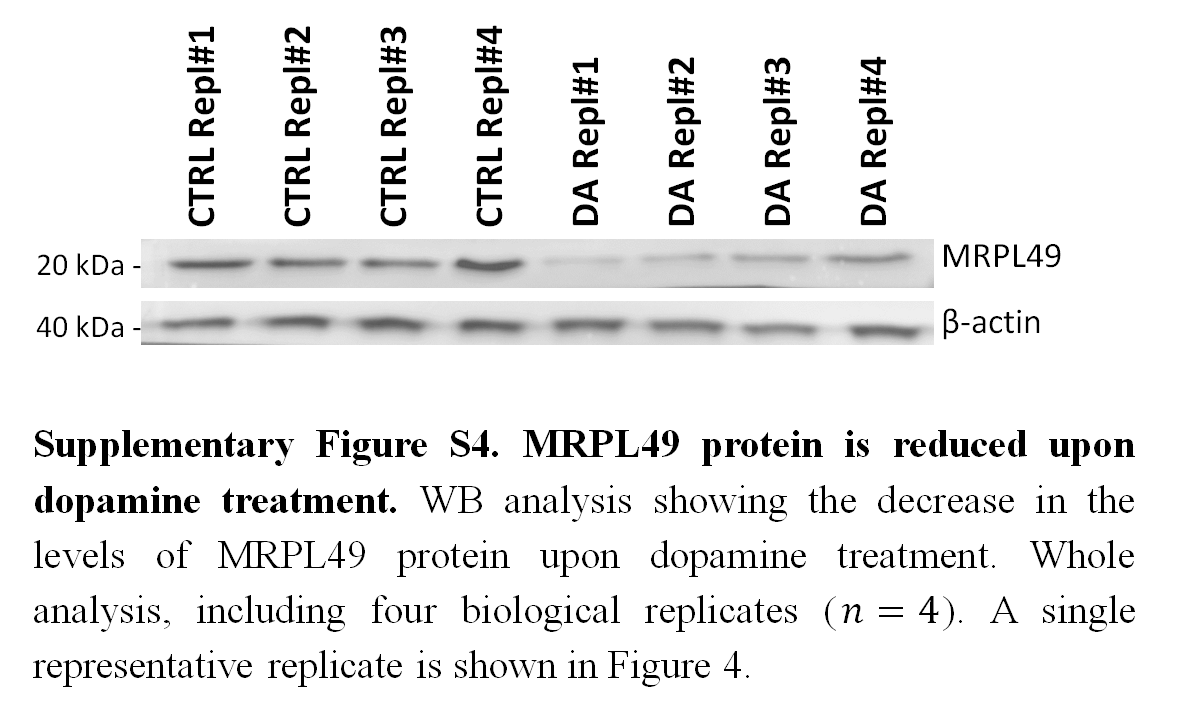

Supplement: Supplementary file 10 [file Image_4.tif]

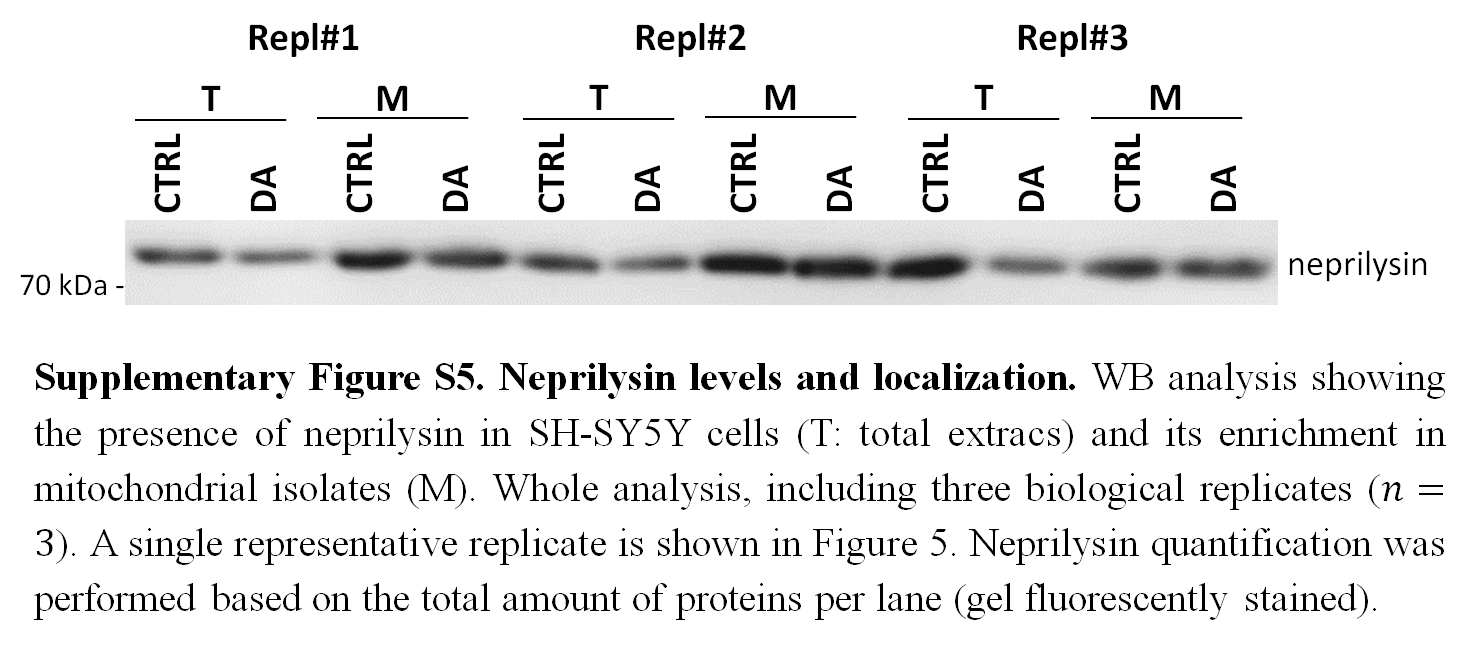

Supplement: Supplementary file 11 [file Image_5.tif]

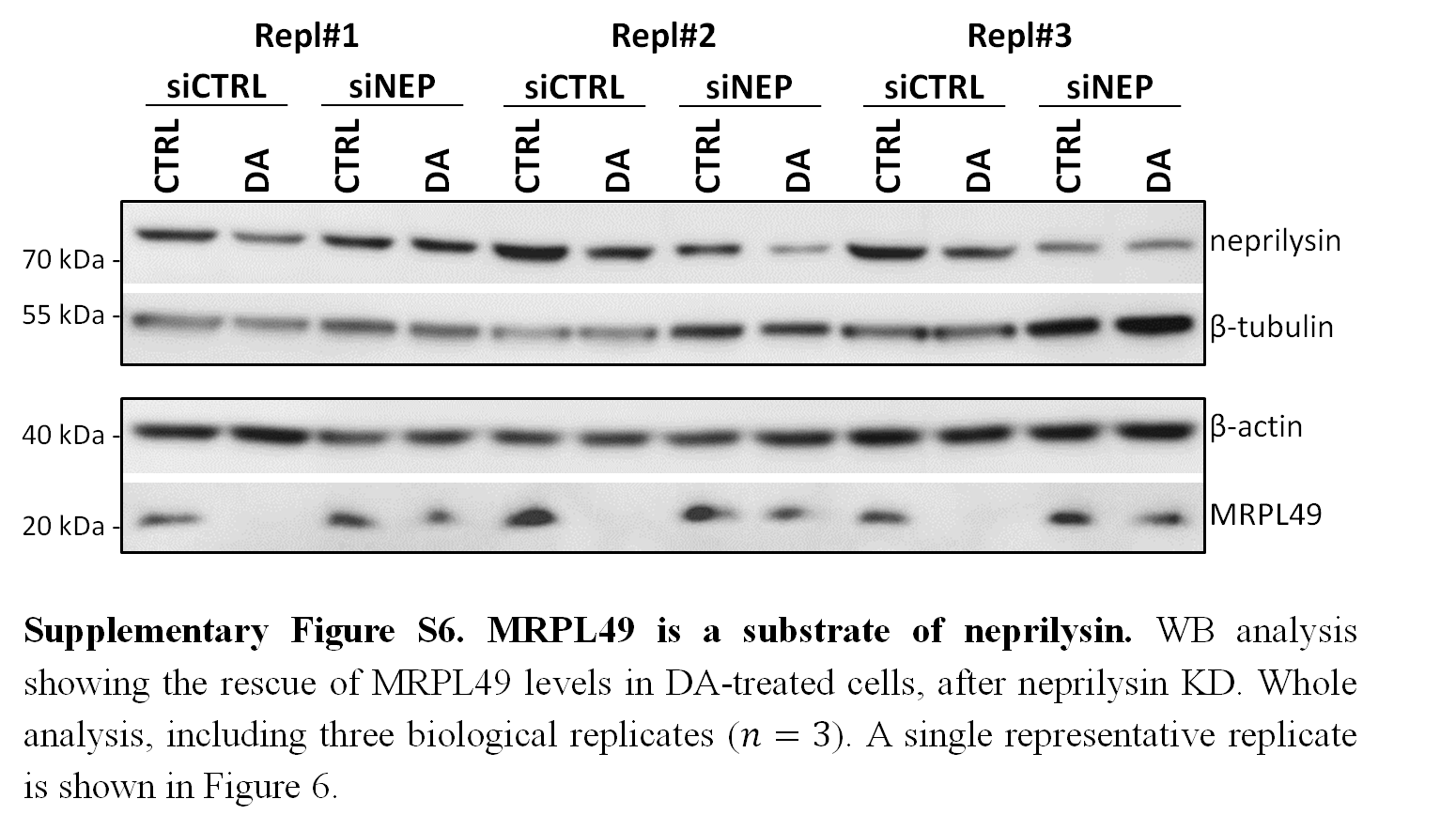

Supplement: Supplementary file 12 [file Image_6.tif]

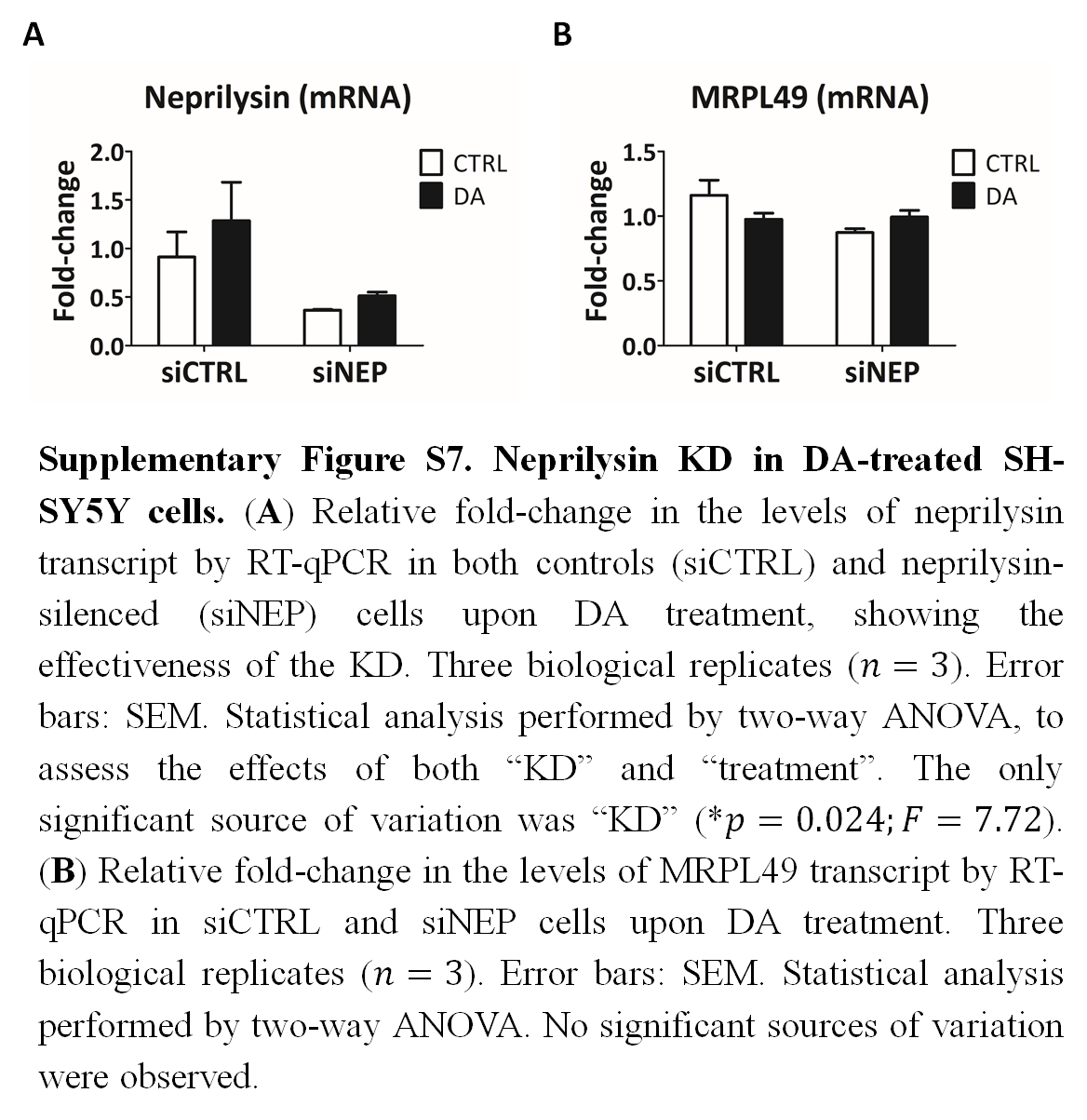

Supplement: Supplementary file 13 [file Image_7.tif]
